# 

**Published:** 1887-04-02

**Authors:** 


### CONTENTS.

**A Little Holiday in the Spring** - - - - 1

**Words of Consolation.**—XXVII. Death - - - 2

**Poetry.**—The Children's Jubilee Hymn - - - 2

**An Italian Quack Doctor at Home.** By an Eye-witness - - - - - - - - 3

**Nurse Marion's Romance.** By Kate Furley. Chapter II. Marion's Home - - - - - 4

**A National Pension Fund for Hospital Officials and Nurses.** By the Secretary of St. Mary's Hospital, the Matron of Oldham Infirmary, etc, - 5

**Public and Private Nursing Institutions.** By a Hospital Physician, and others - - - 6

**Notes and News.**—Items.—The Prince of Wales and the Victoria Hospital for Children.—Tooth Powders.—The East London Nursing Society.—Jubilee Dinners.—Amputating under Difficulties, etc., etc. - - - 8

**Annotations.**—Endowed Beds.—Aberdeen and its Hospitals.—Chronic Hospitals - - - - 11

**Reminiscences of the Insane.** By a Mental Physician - - - - - - - 12

**The Brabazon Home of Comfort.** By Annie Cazenove - - - - - - - 13

**Till the Doctor Comes.**—XIV. Every-day Ailments 14

**Flowers, Ferns, and Ward Decorations** - 15

**Questions and Answers.**—Wanted, Employment as a Nurse in New Zealand.—Wanted, a Home for an Invalid Nurse.—Wanted, Partial Employment for a Trained Nurse.—Cottage Hospital Construction.—Nearly an Idiot - - - - - - 15

**In- and Out-Patients' Hospital Diary** - - 16

**The Children's Corner.**—Puzzles, Answers, etc. - v

**Amusements with Profit** - - - - - v

